# Effects of TIMP1 rs4898 Gene Polymorphism on Early-Onset Preeclampsia Development and Placenta Weight

**DOI:** 10.3390/diagnostics12071637

**Published:** 2022-07-05

**Authors:** Aleksandra E. Mrozikiewicz, Grażyna Kurzawińska, Agata Goździewicz-Szpera, Michał Potograbski, Marcin Ożarowski, Tomasz M. Karpiński, Magdalena Barlik, Piotr Jędrzejczak, Krzysztof Drews

**Affiliations:** 1 Department of Obstetrics and Women’s Diseases, Poznan University of Medical Sciences, Polna 33, 60-535 Poznan, Poland; a.mrozikiewicz@gmail.com; 2 Division of Perinatology and Women’s Diseases, Poznan University of Medical Sciences, Polna 33, 60-535 Poznan, Poland; gene@gpsk.ump.edu.pl (G.K.); agata.szpera@gmail.com (A.G.-S.); m.potograbski@gmail.com (M.P.); kdrews@gpsk.ump.edu.pl (K.D.); 3 Laboratory of Molecular Biology in Division of Perinatology and Women’s Diseases, Poznan University of Medical Sciences, Polna 33, 60-535 Poznan, Poland; 4 Department of Biotechnology, Institute of Natural Fibres and Medicinal Plants, Wojska Polskiego 71b, 60-630 Poznan, Poland; marcin.ozarowski@iwnirz.pl; 5 Department of Medical Microbiology, Poznan University of Medical Sciences, Wieniawskiego 3, 61-712 Poznan, Poland; 6 Independent Researcher, Szamarzewskiego 44D, 60-552 Poznan, Poland; magda.barlik@op.pl; 7 Department of Infertility and Reproductive Endocrinology, Poznan University of Medical Sciences, Polna 33, 60-535 Poznan, Poland; piotr.jedrzejczak@ump.edu.pl

**Keywords:** preeclampsia, metalloproteinases, TIMP1 rs4898 gene polymorphism

## Abstract

Introduction: Some evidence indicates that the improper trophoblast invasion of maternal spiral arteries could be caused by an imbalance between matrix metalloproteinases (MMPs) and tissue inhibitors of metalloproteinases (TIMPs), leading to preeclampsia (PE) development. This study aimed to assess the potential role of *MMP1*, *MMP9*, *TIMP1* and *TIMP2* gene polymorphisms in the pathogenesis of PE. Materials and methods: A total of 308 Polish women, 115 preeclamptic (55 with early-onset preeclampsia [EOPE], 60 with late-onset preeclampsia [LOPE]) and 193 healthy pregnant women, all of Caucasian origin, were recruited to the study. PE was diagnosed following the ACOG criteria. The polymorphic variants of the MMP-TIMP pathway (*MMP1 rs1799750*, *MMP9 rs17576*, *MMP9 rs17577*, *TIMP1 rs4898*, *TIMP2 rs2277698*, *TIMP2 rs55743137*) were genotyped by polymerase chain reaction and restriction fragment length polymorphism. Results: Analyzing all SNPs in the MMP-TIMP pathway, no significant differences in allele frequencies between preeclamptic women and controls were observed. However, comparing the EOPE and LOPE groups with each other, we observed a statistically significant difference between them for the *TIMP1 rs4898* variant. In the whole group of 308 women, the mean placenta weight was the lowest in carriers of the rs4898 CC genotype. Post hoc analysis revealed significant differences between CC-CT (*p* = 0.0209) and CC-TT (*p* = 0.0469). Additionally, during allele analysis, a statistically significant difference in the mean placenta weight (for C allele 529.32 ± 157.11 g, for T allele 560.24 ± 162.24 g, *p* = 0.021) was also observed. Conclusion: Our findings suggest a relationship between TIMP1 rs4898 (372T > C) polymorphism and increased risk of early-onset preeclampsia in a population of pregnant Polish women. Our data suggest that the TIMP1 rs4898 C allele might be associated with increased risk for early-onset, but not for late-onset preeclampsia. To evaluate the role of the TIMP1 polymorphic variants in the etiopathology of preeclampsia, further studies with a larger sample size are needed.

## 1. Introduction

Preeclampsia (PE) is known as one of the most threatening complications of pregnancy and can be associated with fetal growth restriction, preterm delivery, placental abruption and increased fetal and maternal mortality. Preeclampsia complicates 2–8% of pregnancies [1]. Despite intensive research, the pathophysiology of preeclampsia is still discussed and probably multifactorial. The main causative factor is improper placentation of the trophoblast, which further leads to endothelial dysfunction and abnormal morphology of spiral arteries. There is some evidence that improper trophoblast invasion of maternal spiral arteries can be caused by an imbalance between matrix metalloproteinases (MMPs) and tissue inhibitors of metalloproteinases (TIMPs) [2].

MMPs are a family of 28 endopeptidases that are involved in the degradation of extracellular matrix (ECM) components and therefore play a crucial role in physiological as well as pathophysiological tissue remodeling. The MMPs’ activity is suppressed by various endogenous inhibitors, of which the main ones are TIMPs, a family comprising four members (TIMP 1, 2, 3 and 4). MMPs as well as TIMPs play different roles in many physiological processes related to human reproduction, such as ovulation, blastocyst implantation, embryogenesis, and placentation [3,4,5,6]. It is well known that MMP1 and MMP9 are primarily inhibited by TIMP1, and TIMP2 is a crucial inhibitor of MMP2, but it also inhibits, to a lesser extent, MMP9 [7,8].

In early pregnancy, there are activated vasculogenesis, angiogenesis, vascular remodeling and trophoblast invasion, all of these being essential for proper placenta development [9]. The remodeling of the spiral arteries as well as trophoblast invasion may be modulated by proteolysis in the ECM interface and, therefore, these processes may contribute to decidual remodeling at the mother–fetal interface [5]. MMP2 and MMP9 are able to degrade ECM components, especially collagen I and IV, and probably they could be involved in the remodeling of the placenta and uterine artery in healthy pregnancy. Several studies have shown that elevated levels of MMP1, MMP2, and MMP9 are critical for vasodilation, placentation and uterine expansion during healthy pregnancy. Hence, the regulation of the expression of the MMP-TIMP system plays a key role in the development of a normal pregnancy [2].

The abnormal conversion of the spiral arteries may lead to certain complications in pregnancy, including fetal growth restriction and preeclampsia [10]. Decreased vascular MMP1 and MMP9 activity may lead to impaired collagen type I and IV turnover that could result in increased vasoconstriction and decreased blood flow, followed by the development of hypertensive abnormalities in pregnancy [11]. The endothelial cells from preeclamptic placentas produce a smaller amount of MMP1 compared to healthy placentas. MMP imbalance may lead to trophoblast cell apoptosis and reduced uterine perfusion pressure. All these factors are associated with the pathophysiology of preeclampsia. In a few clinical studies, MMP and TIMP level changes were described in women with preeclampsia. Montagnana et al. [12] observed elevated serum levels of TIMP1 in women with preeclampsia compared to healthy pregnant and non-pregnant women, as well as higher levels of TIMP2 in women with pre-eclampsia and healthy pregnant women compared to non-pregnant women [12].

It has also been suggested that MMP and TIMP family genes can play a role in pathophysiology of various cardiovascular diseases such as ischemic stroke, aortic diseases atherosclerosis and preeclampsia [4]. Hence, there is a unique pattern of involvement of *MMP* and *TIMP* genes in pathogenetically different phenotypes. Some studies have investigated *MMP* and *TIMP* gene polymorphisms as predisposing factors for cardiovascular disorders and pregnancy complications [13]. Increased activity of MMPs was found to be associated with aortic diseases and an association was observed between *MMP9* gene polymorphisms (−1562C>T) and risk of ischemic stroke [4]. Several case-control studies have investigated the role of *MMP* gene polymorphisms in preeclampsia [14,15,16,17,18,19]. The most frequently tested polymorphisms were located in the *MMP9* gene (*rs3918242*, *rs2234681*) and the *MMP2* gene (*rs243865*, *rs2285053*). Moreover, two studies were conducted for *TIMP1* and *TIMP3* gene polymorphisms in preeclampsia and showed the association for *TIMP1 rs20705558* and *TIMP3 rs80272* polymorphisms [20,21].

This study aimed to assess the potential role of *MMP1*, *MMP9*, *TIMP1* and *TIMP2* gene polymorphisms in the pathogenesis of preeclampsia. Considering the involvement of these genetic variants in abnormal placentation processes typical for hypertensive disorders during pregnancy, we determined them in healthy pregnant as well as in preeclamptic women.

## 2. Materials and Methods

### 2.1. Study Population

A total of 308 Polish women, 115 preeclamptic and 193 healthy pregnant women, all of Caucasian origin, were recruited to the study. The patients were hospitalized over the years 2018–2021 in the Division of Perinatology and Women’s Diseases, Poznan University of Medical Sciences, Poland. Preeclampsia was diagnosed following the ACOG criteria (disease occurrence after 20 weeks of gestation, systolic blood pressure equal to or higher than 140 mmHg, diastolic blood pressure equal to or higher than 90 mmHg, possible proteinuria presence, other signs of preeclampsia) [22]. The whole group of preeclamptic patients was divided into two subgroups: early-onset preeclampsia (EOPE) that develops before 34 week of gestation (*n* = 55) and late-onset preeclampsia (LOPE) that occurs after 34 weeks of gestation (*n* = 60). We applied the following criteria for exclusion from the study groups: multiple gestations, fetal structural/genetic anomaly, maternal diseases such as diabetes mellitus, chronic hypertension, as well as renal, endocrinological and autoimmune diseases.

In the control group we enrolled healthy normotensive pregnant women without any fetal disorder or multiple pregnancies. All patients were informed about the goal of the study and gave their written consent. The study was approved by the Local Bioethical Committee at Poznan University of Medical Sciences (493/21).

### 2.2. DNA Extraction

Peripheral venous blood samples were collected from case and control groups into sterile EDTA coated tubes. Genomic DNA extraction was performed using the DNA Blood Mini Kit (Qiagen, Hilden, Germany) according to the manufacturer’s protocol. The amount of the total extracted DNA was determined using the spectrophotometric method (NanoDrop Spectrophotometer, Thermo Scientific, Waltham, MA, USA). The samples were stored at −20 °C until analysis.

### 2.3. Genotyping

The polymorphic variants mentioned in Table 1 were genotyped by polymerase chain reaction and restriction fragment length polymorphism (PCR-RFLP). PCR amplification was carried out in a final volume of 25 µL using a DNA Engine Dyad Thermocycler (Bio-Rad Laboratories, Inc., Hercules, CA, USA). The reaction mixture consisted of 100 ng of genomic DNA, 0.25 µM of each primer (TiBMolBiol, Berlin, Germany), 1 U of Taq DNA polymerase and respective buffer (DreamTaq Green DNA polymerase, Thermo Fisher Scientific, Waltham, MA, USA), 200 µM dNTP Mix (Thermo Fisher Scientific, Waltham, MA, USA) and deionized water. PCR reaction conditions were optimized for each polymorphism. Characteristics of the polymorphisms and the specific primer sequences are shown in Table 2. Amplified fragments were then digested with the appropriate restriction enzyme (Thermo Fisher Scientific, Waltham, MA, USA), according to the manufacturer’s instructions, and visualized after electrophoresis on 2% or 3% agarose gel with Midori Green Advanced DNA Stain (Nippon Genetics, Europe GmbH, Düren, Germany).

### 2.4. Statistical Analyses

All statistical analyses were conducted in the R statistical software version 4.0.3 [23] and ggstatsplot package [24]. Quantitative data are presented as mean ± SD, whereas frequency and percentages were used for qualitative variables. Differences were calculated by chi-square and Fisher exact tests (for categorical variables) or the Student *t*-test (for continuous variables). The Hardy–Weinberg equilibrium (HWE) was assessed using a χ^2^ test in each group. Genotype and allele frequency distributions were determined under the different models of inheritance (codominant, dominant, recessive) using the SNPassoc package [25]. ANOVA and the post-hoc Tukey method were carried out for a comparison of genotypes and clinical characteristics. Haplotype analysis was performed using the haplo.stats package [26]. A *p* value less than 0.05 was considered significant. Additionally, we used the nonparametric multifactor dimensionality reduction (MDR, v3.0.2) approach to detect potential gene–gene interactions. To minimize the chance of obtaining type I errors, the false discovery rate (FDR) approach was used. GAS (Genetic Association Study Power Calculator) (https://csg.sph.umich.edu/abecasis/gas_power_calculator/index.html accessed on 29 June 2022) was used for post-hoc power calculation. After considering sample size (cases = 115 and controls = 193), preeclampsia prevalence 2–8% [1]—mean 5%, MAF, and significance level=0.05, the overall study power (average of six tested variants) was calculated at 62.0%.

## 3. Results

### 3.1. Clinical Characteristics of the Study Population

The selected characteristics of the study women are presented in Table 3. A total of 115 women at 25–40 weeks of gestation with preeclampsia were enrolled in this study. The differences in clinical parameters between the study and control groups were mostly significant, except for the mother’s age and sex of the newborn. The mean maternal age for PE group was 30.32 ± 5.32 years and for healthy subjects it was 31.06 ± 3.89 (*p* = 0.2019). Among the patients with preeclampsia, 55 (47.83%) were diagnosed before 34 weeks of gestation, and 60 (52.17%) patients were diagnosed at or after 34 weeks of gestation. No significant difference was found in age, diastolic blood pressure, pre- and post-pregnancy BMI, number of pregnancies or infant sex between early and late onset preeclampsia (*p* > 0.05 for each variable; Table 3). In the next stage, we compared the laboratory outcomes between early- and late-onset preeclampsia. There were no significant differences between groups in urea, uremic acid, creatinine and alanine and aspartate transaminase concentrations. Women in the EOPE group were characterized by a significantly higher level of proteinuria (369.96 ± 181.79 mg/dL vs. 194.37 ± 164.78 mg/dL, *p* < 0.001), higher 24-h urine proteinuria (4.86 ± 2.77 g/24 h vs. 2.49 ± 3.42 g/24 h, *p* = 0.0499) and lower levels of total protein in the blood compared with the LOPE group (5.47 ± 0.57 g/dL vs. 5.85 ± 0.78, *p* = 0.0049) (Table 4).

### 3.2. Association Analysis of MMP1, MMP9, TIMP1 and TIMP2 Polymorphisms

For all SNPs, the genotype distribution in PE and controls was in the Hardy–Weinberg equilibrium (*p* > 0.05). No significant differences were observed in allele frequencies between preeclamptic women and controls (Table 5). The largest differences in the frequency of alleles were observed for *TIMP1 rs4898*. Allele C was observed in 47% of LOPE women, 50.0% of controls, 53% of PE and 60.0% of the EOPE group (EOPE vs. control: OR = 1.5, *p* = 0.063, EOPE vs. LOPE: OR = 1.7, 95%CI = 1.02–2.89, *p* = 0.042, p_cor_. = 0.257).

Table 6 presents the single-locus analysis for the six investigated SNPs within the *MMP1*, *MMP9*, *TIMP1* and *TIMP2* genes in PE and control women. None of the polymorphisms were associated with the PE risk under the codominant, dominant and recessive models (also after adjustment for pre-pregnancy BMI). Next we evaluated the associations among studied variants when the EOPE and LOPE groups were compared with each other and with the control group. Genotype distributions of polymorphism in EOPE and LOPE subtypes are given in Table 7. Analysis of genotype distributions revealed a non-significant difference for *TIMP1 rs4898* polymorphism between the EOPE group and controls in the dominant model (CC vs. CT + TT) (0.665 vs.0.777, OR = 1.84, 95% CI: 0.96–3.53, *p* = 0.0706). Our data indicated no significant difference in the genotype frequencies of studied polymorphisms between late-onset preeclampsia and control groups. However, comparing EOPE and LOPE groups with each other, we observed a statistically significant difference between them for the *TIMP1 rs4898* variant. In the recessive model the CC + CT genotype frequency was 85.5% in the EOPE and 70.0% in the LOPE subgroup (OR = 2.52, 95% CI: 0.99–6.39, *p* = 0.0452).

### 3.3. Haplotype and Gene-Gene Interaction Analysis

#### Haplotype Analysis of the MMP9 and TIMP2 Gene Polymorphisms

The studied *MMP9* and *TIMP2* polymorphisms were further characterized using linkage disequilibrium (LD) and haplotype analyses. Interaction of these variants within haplotypes can be more informative than single-locus analysis. A linkage disequilibrium study showed for the *MMP9* gene variants D’ = 0.93 (r^2^ = 0.29) and for *TIMP2* variants D’ = 0.91 (r^2^ = 0.61).

The estimated haplotype frequencies for the two MMP9 and two TIMP2 polymorphisms are shown in Table 8. Similar to the genotype analysis, there were no significant differences between groups in the distributions of haplotype frequencies when PE, EOPE and LOPE were compared with the control group.

**Figure 1:**
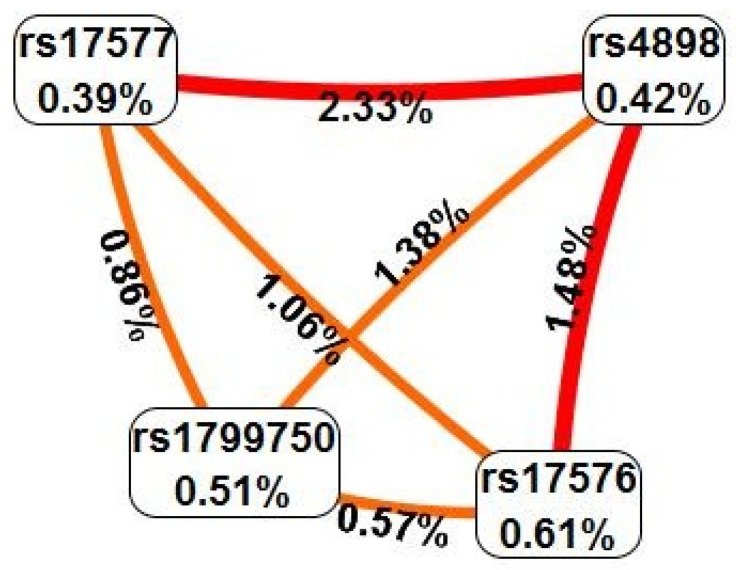
Interaction entropy graph obtained by multifactor dimensionality reduction for matrix metalloproteinases and tissue inhibitor of metalloproteinases in preeclampsia.

To explore epistatic interactions, we assessed all pairwise gene–gene interactions using the SNPassoc R CRAN package and MDR v3.0.2 software. Prominent gene–gene interactions were detected between *TIMP1* (*rs4898*) and *MMP9* (*rs17577*) under codominant (*p* = 0.0388) and over-dominant (*p* = 0.0145) models. An interaction was also detected between *TIMP1* (*rs4898*) and *MMP9* (*rs17576*) only under over dominant model (*p* = 0.0388). However, the *p* values of interactions were not significant after correction for multiple tests. The entropy-based interaction graph provided with MDR software shows the most significant gene-gene models for the four SNPs (*rs17576*, *rs17577*, *rs4898*, and *rs17997500*). The graph suggests that these interactions are interdependent, and synergism was observed between the polymorphisms of the TIMP1 and MMP9 genes (Figure 1).

### 3.4. Association between Genotypes and Clinical Characteristics

In the next stage, we examined the association between genotypes and clinical data of the studied women. The *TIMP1* gene variant rs4898 was associated in all study women with placenta weight. The mean placenta weight for the PE group was 423.53 ± 162.68 g (range 100.00–900.00 g) and for healthy subjects 611.30 ± 113.43 g (range 330.00–980.00 g) (*p* < 0.001).

**Figure 2:**
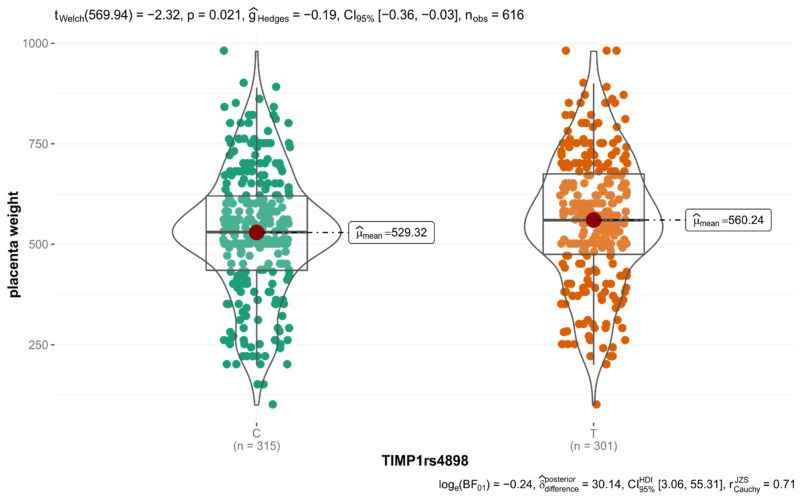
Association between TIMP1 rs4898 alleles and placenta weight in all studied women.

In the whole group of 308 women, the mean weight of the placenta was the lowest in carriers of the *rs4898* CC genotype (496.84 ± 145.45 g for CC genotype, 558.62 ± 162.30 g for CT and 562.15 ± 163.42 g for TT, respectively; *p* = 0.0172 in the codominant model and *p* = 0.0044 in the dominant model). Post hoc analysis revealed significant differences between CC-CT (*p* = 0.0209) and CC-TT (*p* = 0.0469). Additionally, during allele analysis, a statistically significant difference between the mean weights of the placenta (for C allele 529.32 ± 157.11 g, for T allele 560.24 ± 162.24 g, *p* = 0.021) was observed (Figure 2).

## 4. Discussion

Pre-eclampsia is a pregnancy-specific disease in which some genetic aspects may play a significant role in its development [27]. In the literature, various genetic polymorphisms related to pre-eclampsia have been described [28,29]. The MMPs play an important role in morphogenesis, angiogenesis and tissue repair, mainly through tissue remodeling. Tissue inhibitors of metalloproteinases (TIMPs) are endogenous inhibitors of MMPs and are consequently significant regulators of ECM turnover, as well as tissue remodeling. Dysregulation of the balance between MMPs and TIMPs is characteristic for some acute and chronic cardiovascular diseases [30]. Moreover, some authors have reported a relationship between MMP gene polymorphism and hypertensive disorders in pregnancy. However, most of them did not detect an association between different MMP variants and preeclampsia or gestational hypertension development.

The aim of the study by Rahimi et al. [31] was to investigate the possible influence of two polymorphisms—*MMP7* −181A>G and *MMP9* −1562C>T—on the risk of preeclampsia and lipid peroxidation level. The frequency of the *MMP7 −*181G allele was not significantly different between the groups with severe and mild preeclampsia as well as the healthy pregnant group. This study did not reveal a direct influence of *MMP7 −*181A>G polymorphism on the risk of preeclampsia. On the other hand, this polymorphism, through elevation of MDA level and interaction with *MMP9 −*1562C>T polymorphism, might be associated with the risk of severe preeclampsia [31].

Other research analyzed the relationship between *MMP2* polymorphisms *(−306C>T* and −735C>T) and the response to antihypertensive treatment in a preeclamptic group, patients with gestational hypertension and healthy pregnant women. This study showed that *MMP2* polymorphisms do not influence the development of hypertensive disorders of pregnancy and additionally do not affect the responsiveness to antihypertensive therapy in hypertensive disorders of pregnancy, especially preeclampsia [32].

In our study we also did not find any association between genetic polymorphism in genes encoding *MMP1* and *MMP9* and risk of preeclampsia. The genotype results obtained in our research confirm previous findings which show similar distribution of genetic variants in healthy pregnant and preeclampsia groups.

Fraser et al. reported the lack of a significant association of *MMP9 −*1562C>T polymorphism with preeclampsia risk and concluded that this *MMP9* variant has little value as a screening marker for susceptibility to the syndrome [33].

Coolman et al. [14] reported that the frequency of the *MMP9 −*1562T allele was 0.06 in women with preeclampsia and 0.12 in healthy pregnant patients. Additionally, women carrying the T allele were less likely to develop preeclampsia than women with the CC genotype. However, they did not detect any association between presence of the T allele (CT + TT genotypes) and severe preeclampsia, eclampsia or HELLP syndrome [14]. Another study reported no statistically significant differences in the frequencies of the *MMP1* genotypes between preeclamptic patients and a healthy pregnant control group (*p* = 0.9137). Allele frequencies did not exhibit any significant differences between study and control groups (*p* = 0.4210) or among subgroups (mild/severe preeclampsia, eclampsia or chronic hypertension with superimposed preeclampsia) [34].

The lack of significant associations between genotypes for the polymorphisms and preeclampsia suggests no association between susceptibility to this disease and genetic variations in *MMP1*, *MMP9*, *TIMP1* and *TIMP2*. It might be suspected that not maternal but fetal genotype affects the susceptibility to preeclampsia, and this issue should be addressed in further studies.

In contrast to the above-mentioned results, Sun et al. [19] found in pregnant Chinese women that *MMP9 −*1562C>T genetic polymorphism might be associated with the development of pre-eclampsia. Women carrying the T allele were at higher risk of pre-eclampsia compared to women carrying the C allele (OR = 1.62) [19].

Additionally, Palei et al. [15,16] found an association between *MMP9 −*1562C>T polymorphism and gestational hypertension, but not with preeclampsia [15,16]. Additionally, *MMP9* haplotypes (*−*1562C>T/*−*90(CA)13–25) affect the responsiveness to antihypertensive therapy of gestational hypertension [16].

TIMP1 is a natural inhibitor of MMPs’ activity and limits their proteolytic activities against the ECM. In consequence, it may affect the process of pregnancy vascular remodeling [35,36]. Moreover, genetic polymorphism of TIMP-1 has been linked with higher risk of developing certain diseases such as essential hypertension or intracerebral hemorrhage, which share certain risk factors with preeclampsia [13,37].

Our data did not reveal a significant difference in genotype frequencies of studied polymorphisms between LOPE/EOPE and control groups. However, what is very interesting is that, comparing subgroups EOPE and LOPE with each other, we observed a statistically significant difference between them in frequency of the *TIMP1 rs4898* variant. The stratified analysis showed in the recessive model CC + CT genotype frequency 85.5% in the EOPE and 70.0% in the LOPE subgroup (OR = 2.52, *p* = 0.0452) which suggests that *TIMP1 rs4898* could be associated with the form of preeclampsia. This observation might indicate that carriers of the *TIMP1 rs4898* C allele represent a select group of patients who need, even in early pregnancy, close monitoring for the first symptoms of preeclampsia.

Additionally, in our analysis the same polymorphic variant *TIMP1 rs4898* affected placental weight in all study women. In the whole group of 308 women, the lowest placenta weight was observed in carriers of *TIMP1 rs4898* CC genotype (*p* = 0.0172 in the codominant model and *p* = 0.0044 in the dominant model). Recently, it was shown that *TIMP1* polymorphism may possibly influence *TIMP1* expression or modify its biological properties. Moreover, the *TIMP1 rs4898* T allele reduces the activity of the TIMP1 protein, resulting in higher MMP activity, and provides normal angiogenesis and a normal placenta in carriers of this variant [13]. This may be associated with higher placental weights in carriers of the CT and TT genotypes compared to those of the CC genotype.

Due to the small number of studies concerning the involvement of *TIMP* genetic polymorphism in PE development, definite conclusions require future research. Nevertheless, the role of TIMP1 genetic polymorphisms in the etiopathogenesis of PE was demonstrated in the study of Luizon et al. [38]. The authors found that *TIMP1* −9830T>G polymorphism is associated with preeclampsia (association with PE in carriers of the G allele as well as the GG genotype). Additionally, TIMP1 levels were higher in preeclamptic patients with the *TIMP1* −9830TG genotype. Their data also suggested that this polymorphism may be implicated in poor responses to antihypertensive drugs [38].

### Study Limitations

Obviously, there are some limitations of our study. Firstly, there is a small number of patients in the study group, but it should be noted that the sample was collected for a case–control study, which ensured the high quality of analysis. The post-hoc power analysis was performed using the overall study power (average of six tested variants) was under-powered, calculated at 62.0%. However, it is possible that studying a greater number of subjects would allow us to detect other significant associations in the MMP-TIMP pathway. Besides the sample size, the limitations of the research are also the restrictions due to race and region. Secondly, we focused on certain polymorphisms in the MMP-TIMP pathway, but it seems that the selected variants of the *MMP1*, *MMP9*, *TIMP1*, *TIMP2* genes could play an important role in extracellular matrix remodeling. Consequently, an important future task is to establish the influence of MMP polymorphisms on enzyme activities as well as the exact interaction between MMPs and TIMPs. 

On the other hand, it could be also interesting to determine, besides the analysis of genetic polymorphisms of the MMPs-TIMPs system, the expression level of these genes. Additionally, we have also consciousness that the preeclampsia may be related to the above genes in the fetus, thus it would be advisable to study these genes in the mother–child system as well. 

Furthermore, we noted that there are differences between our results and those previously published by other authors. This could be due to the number and ethnicity of patients, the preeclampsia definition, or the methodology of MMP and TIMP detection.

## 5. Conclusions

The present results indicate a relationship between *TIMP1 rs4898* (372T > C) polymorphism and increased risk of early-onset preeclampsia (EOPE) in a population of pregnant Polish women. Our data suggest that the *TIMP1 rs4898* C allele might be associated with increased risk for early-onset, but not late-onset, preeclampsia. This finding could suggest that *TIMP* polymorphisms actively modulate ECM remodeling in the early stage of trophoblast invasion, and disturbances in this process result in preeclampsia development. The active participation of *TIMP* polymorphisms in ECM placenta remodeling was also confirmed by our secondary observations, indicating that the *TIMP1 rs4898* C allele is associated with a lower placenta weight in the studied population of Polish women.

Although there is quite a lot of evidence for the importance of the MMP-TIMP pathway in trophoblast invasion, the aspects concerning the molecular mechanism remain unclear. To evaluate the role of the *TIMP1* polymorphic variants in the etiopathology of preeclampsia, further studies with larger sample sizes and in other populations are needed.

## Figures and Tables

**Table 1:** Characteristics of selected variants in genes MMP1, MMP9, TIMP1 and TIMP2.

Gene	*MMP1*	*MMP9*		*TIMP1*	*TIMP2*	
*rs number*	*rs1799750*	*rs17576*	*rs17577*	*rs4898*	*rs2277698*	*rs55743137*
*Chromosome position (GRCh38.p12)*	chr11:102799765-102799766	chr20:46011586	chr20:46014472	chrX:47585586	chr17:78870935	chr17:78871103
*Traditional name*	−1607 1G/2G	836A > G	2003G > A	372T > C	303C > T	—
*Genomic positions* *	NG_011740.2: g.3471del	NG_011468.1: g.7679A > G	NG_011468.1: g.10565G > A	NG_012533.1: g.8296T > C	NC_000017.11: g.78870935C > T	NC_000017.11: g.78871103G > T
*Alleles*	delC	A > G	G > A	T > C	C > T	G > T
*Amino acid change*	none	Q279R	R668Q	F124=	S101=	none
*Location in gene*	2 KB Upstream Variant	Exon 6	Exon 12	Exon 5	Exon 3	Intron 2

GRCh38 is the Genome Reference Consortium Human genome build 38 patch release 12. * Human Genome Variation Society (HGVS).

**Table 2:** Primer sequences, annealing temperatures, and restriction enzymes of the polymorphisms.

Gene rs Number	Primer Sequence	Amplicon Size	Restriction Enzyme	RFLP Product Size
*MMP1* *rs1799750*	5′-TGACTTTTAAAACATAGTCTATGTTCA-3′ 5′-TCTTGGATTGATTTGAGATAAGTCATAGC-3′	269 bp	AluI	1G 241, 28 bz 2G 269 bz
*MMP9* *rs17576*	5′-GAGAGATGGGATGAACTG-3′ 5′-GTGGTGGAAATGTGGTGT-3′	439 bp	MspI	A 252, 187 bz G 187, 129, 123 bz
*MMP9* *rs17577*	5′-ACACGCACGACGTCTTCCAGTATC-3′ 5′-GGGGCATTTGTTTCCATTTCCA-3′	138 bp	TaqI	G 115, 23 bz A 138 bz
*TIMP1* *rs4898*	5′-GCACATCACTACCTGCAGTCT-3′ 5′-GAA ACA AGC CCA CGA TTT AG-3′	175 bz	BauI (BssSI)	T 175 bz C 153, 22 bz
*TIMP2* *rs2277698*	5′-CCAGGAAATTGGCAGGTAGT-3′ 5′-GAATTCACCAACTGTGTGGC-3′	369 bz	BsrI	C 369 bz T 231, 138 bz
*TIMP2* *rs55743137*	5′-CCTTTGAACATCTGGAAAGACAA-3′ 5′-TAACCCATGTATTTGCACTTCCT-3′	160 bz	AluI	T 160 pz G 108, 52 bz

**Table 3:** Selected clinical characteristics of the PE and healthy women in the association study.

Variables	PE (*n* = 115)	Controls (*n* = 193)	*p*	EOPE (*n* = 55)	LOPE (*n* = 60)	*p*
Maternal age (years)	30.32 ± 5.32	31.06 ± 3.89	0.2019	30.11 ± 5.22	30.53 ± 5.45	0.6782
Gestational age (weeks)	33.69 ± 3.58	38.97 ± 1.09	<0.001	30.56 ± 2.10	36.61 ± 1.71	<0.001
Systolic blood pressure (mmHg)	171.71 ± 18.63	104.56 ± 10.85	<0.001	175.64 ± 19.51	168.05 ± 17.15	0.0335
Diastolic blood pressure (mmHg)	106.80 ± 12.81	66.01 ± 7.77	<0.001	107.34 ± 13.53	106.20 ± 12.18	0.6157
Pre-pregnancy BMI (kg/m^2^)	25.17 ± 5.83	21.00 ± 2.50	<0.001	24.99 ± 4.67	25.34 ± 6.74	0.7534
Post-pregnancy BMI (kg/m^2^)	30.48 ± 5.27	26.17 ± 2.94	<0.001	30.16 ± 4.12	30.78 ± 6.16	0.5225
Caesarean section, N (%)	104 (90.43)	65 (33.68)	<0.001 *	54 (98.18)	50 (83.33)	0.009 #
Primipara, N (%)	69 (60.00)	22 (11.40)	<0.001 *	33 (60.00)	36 (60.00)	1.000 *
Infant sex—girl N (%)	59 (51.30)	81 (41.97)	0.1117 *	28 (50.91)	31 (51.67)	0.9203 *
Infant birthweight (g)	1892.97 ± 857.63	3467.46 ± 409.17	<0.001	1323.15 ± 426.30	2442.81 ± 809.98	<0.001
Apgar score at 1 min	7.35 ± 2.70	9.87 ± 0.47	<0.001	6.11 ± 2.62	8.54 ± 2.21	<0.001
Apgar score at 5 min	8.58 ± 1.54	9.98 ± 0.13	<0.001	7.85 ± 1.63	9.29 ± 1.06	<0.001
Placenta weight (g)	423.53 ± 162.68	611.30 ± 113.43	<0.001	325.64 ± 100.41	517.65 ± 155.88	<0.001

Values are presented as mean ± SD or *n* (%), *p* value—Student’s *t*-test, # Fisher test, * Pearson’s χ^2^, BMI = body mass index.

**Table 4:** Laboratory findings of patients according to onset of preeclampsia.

Variables	All PE Women (*n* = 115)	EOPE (*n* = 55)	LOPE (*n* = 60)	*p* *EOPE* vs. *LOPE*
Urea (mg/dL)	30.23 ± 13.62	32.11 ± 13.58	28.45 ± 13.52	0.1577
Uremic acid (mg/dL)	6.49 ± 1.33	6.74 ± 1.50	6.26 ± 1.10	0.0593
Total protein (g/dL)	5.67 ± 0.71	5.47 ± 0.57	5.85 ± 0.78	0.0049
Creatinine (mg/dL)	0.75 ± 0.21	0.81 ± 0.26	0.69 ± 0.11	0.0919
Proteinuria (mg/dL)	279.79 ± 193.69	369.96 ± 181.79	194.37 ± 164.78	<0.001
ALT, U/L	35.33 ± 43.42	50.69 ± 56.75	20.99 ± 17.98	0.0803
AST, IU/L	36.07 ± 38.62	48.46 ± 50.81	24.51 ± 17.01	0.1131
Proteinuria, g/24 h	3.72 ± 3.27	4.86 ± 2.77	2.49 ± 3.42	0.0499

ALT = alanine transaminase, AST = aspartate transaminase, *p* value—Student’s *t*-test.

**Table 5:** Genotype frequencies of the MMP1, MMP9, TIMP1 and TIMP2 polymorphisms and their association with risk of PE.

SNP	Alleles		HWE *p*	OR (95%CI)	*p*	Allele Frequency in European Population *
*MMP1 rs1799750*	*1G*	*2G*				
*PE*	113 (0.491)	117 (0.508)	0.960	1.274 (0.919–1.768)	0.145	*1G* = 0.4960 *2G* = 0.5040
*control*	213 (0.551)	173 (0.448)	0.997			
*MMP9 rs17576*	*A*	*G*				
*PE*	143 (0.621)	87 (0.378)	0.622	1.157 (0.824–1.624)	0.398	*A* = 0.6193 *G* = 0.3807
*control*	253 (0.655)	133 (0.344)	0.616			
*MMP9 rs17577*	*G*	*A*				
*PE*	289 (0.821)	41 (0.178)	0.103	1.305 (0.839–2.031)	0.236	*G* = 0.8250 *A* = 0.1750
*control*	331 (0.857)	55 (0.142)	0.471			
*TIMP1 rs4898*	*T*	*C*				
*PE*	108 (0.469)	122 (0.531)	0.971	1.129 (0.814–1.566)	0.464	*T* = 0.535 *C* = 0.465
*control*	193 (0.500)	193 (0.500)	0.319			
*TIMP2 rs2277698*	*C*	*T*				
*PE*	196 (0.852)	34 (0.147)	0.545	0.980 (0.62–1.552)	0.999	*C* = 0.8748 *T* = 0.1252
*control*	328 (0.849)	58 (0.151)	0.651			
*TIMP2 rs55743137*	*T*	*G*				
*PE*	181 (0.786)	49 (0.213)	0.992	1.222 (0.812–1.838)	0.343	*T* = 0.8002 *G* = 0.1998
*control*	316 (0.818)	70 (0.181)	0.523			

Pearson’s *p*, * 1000 Genomes Project phase3 release V3+.

**Table 6:** Genotype frequencies of the MMP1, MMP9, TIMP1 and TIMP2 polymorphisms and their association with risk of PE.

SNP	Genotypes	PE (*n* = 115)	Control (*n* = 193)	OR (95%CI)	*p-Value*	* *p-Value*
*MMP1 rs1799750*	*1G*/*1G*	27 (0.235)	59 (0.305)	1.00	0.3332	0.3455
	*1G*/*2G*	59 (0.513)	95 (0.492)	0.74 (0.42–1.29)		
	*2G*/*2G*	29 (0.252)	39 (0.202)	0.62 (0.32–1.19)		
	Dominant	88 (0.765)	134 (0.694)	0.70 (0.41–1.18)	0.1762	0.1548
	Recessive	86 (0.748)	154 (0.798)	0.75 (0.43–1.30)	0.3080	0.4482
*MMP9 rs17576*	*AA*	42 (0.365)	86 (0.446)	1.00	0.2731	0.7135
	*AG*	59 (0.513)	81 (0.420)	0.67 (0.41–1.10)		
	*GG*	14 (0.122)	26 (0.134)	0.91 (0.43–1.91)		
	Dominant	73 (0.635)	107 (0.554)	0.72 (0.45–1.15)	0.1649	0.4127
	Recessive	101 (0.878)	167 (0.865)	1.12 (0.56–2.25)	0.7422	0.8464
*MMP9 rs17577*	*GG*	81 (0.704)	144 (0.746)	1.00	0.4329	0.2268
	*GA*	27 (0.235)	43 (0.223)	0.90 (0.52–1.56)		
	*AA*	7 (0.061)	6 (0.031)	0.48 (0.16–1.48)		
	Dominant	34 (0.296)	49 (0.254)	0.81 (0.48–1.36)	0.4260	0.1754
	Recessive	108 (0.939)	187 (0.969)	0.50 (0.16–1.51)	0.2171	0.1495
*TIMP1 rs4898*	*CC*	33 (0.287)	43 (0.223)	1.00	0.4046	0.7358
	*TC*	56 (0.487)	107 (0.554)	1.47 (0.84–2.56)		
	*TT*	26 (0.226)	43 (0.223)	1.27 (0.65–2.47)		
	Dominant	82 (0.713)	150 (0.777)	1.40 (0.83–2.38)	0.2093	0.4965
	Recessive	89 (0.774)	150 (0.777)	0.98 (0.56–1.71)	0.9466	0.8806
*TIMP2 rs2277698*	*CC*	85 (0.739)	141 (0.731)	1.00	0.9590	0.8303
	*CT*	26 (0.226)	46 (0.238)	1.07 (0.61–1.85)		
	*TT*	4 (0.035)	6 (0.031)	0.90 (0.25–3.30)		
	Dominant	30 (0.261)	52 (0.269)	1.04 (0.62–1.76)	0.8693	0.8443
	Recessive	111 (0.965)	187 (0.969)	0.89 (0.25–3.22)	0.8602	0.6292
*TIMP2 rs55743137*	*TT*	71 (0.617)	127 (0.658)	1.00	0.4767	0.5195
	*TG*	39 (0.339)	62 (0.321)	0.89 (0.54–1.46)		
	*GG*	5 (0.043)	4 (0.021)	0.45 (0.12–1.72)		
	Dominant	44 (0.383)	66 (0.342)	0.84 (0.52–1.35)	0.4724	0.3917
	Recessive	110 (0.957)	189 (0.979)	0.47 (0.12–1.77)	0.2609	0.3510

*p*-values multivariable logistic regression. * *p* value adjusted for BMI before pregnancy; OR—odds ratio, 95% CI—95% confidence interval.

**Table 7:** Genotype distributions of study variants in PE subtypes.

SNP	Genotypes/Models	Controls (*n* = 193) *n* (%)	EOPE (*n* = 55) *n* (%)	LOPE (*n* = 60) *n* (%)	EOPE vs. Control		LOPE vs. Control		EOPE vs. LOPE	
					OR (95%CI)	*p*	OR (95%CI)	*p*	OR (95%CI)	*p*
*MMP1 rs1799750*	1G/1G	59 (0.305)	12 (0.218)	15 (0.250)	1.00	0.4297	1.00	0.5095	1.00	0.8007
	1G/2G	95 (0.492)	30 (0.545)	29 (0.483)	0.64 (0.31–1.36)		0.83 (0.41–1.68)		1.29 (0.52–3.23)	
	2G/2G	39 (0.202)	13 (0.236)	16 (0.267)	0.61 (0.25–1.48)		0.62 (0.28–1.40)		1.02 (0.35–2.92)	
	Dominant	134 (0.694)	43 (0.782)	45 (0.750)	0.63 (0.31–1.29)	0.1963	0.76 (0.39–1.46)	0.4024	1.19 (0.50–2.84)	0.6873
	Recessive	154 (0.798)	42 (0.764)	44 (0.733)	0.82 (0.40–1.67)	0.5855	0.70 (0.36–1.36)	0.2974	0.85 (0.37–1.98)	0.7083
*MMP9 rs17576*	AA	86 (0.446)	21 (0.382)	21 (0.350)	1.00	0.1841	1.00	0.4137	1.00	0.2929
	AG	81 (0.420)	30 (0.545)	29 (0.483)	0.66 (0.35–1.24)		0.68 (0.36–1.29)		0.97 (0.44–2.13)	
	GG	26 (0.134)	4 (0.073)	10 (0.167)	1.59 (0.50–5.04)		0.63 (0.27–1.52)		2.50 (0.68–9.25)	
	Dominant	107 (0.554)	34 (0.618)	39 (0.650)	0.77 (0.42–1.42)	0.3976	0.67 (0.37–1.22)	0.1874	1.15 (0.54–2.45)	0.7234
	Recessive	167 (0.865)	51 (0.927)	50 (0.833)	1.99 (0.66–5.95)	0.1908	0.78 (0.35–1.72)	0.5422	2.55 (0.75–8.67)	0.1176
*MMP9 rs17577*	GG	144 (0.746)	40 (0.727)	41 (0.683)	1.00	0.7361	1.00	0.4283	1.00	0.8727
	GA	43 (0.223)	12 (0.218)	15 (0.250)	1.00 (0.48–2.06)		0.82 (0.41–1.62)		1.22 (0.51–2.93)	
	AA	6 (0.031)	3 (0.055)	4 (0.067)	0.56 (0.13–2.32)		0.43 (0.12–1.59)		1.30 (0.27–6.18)	
	Dominant	49 (0.254)	15 (0.273)	19 (0.317)	0.91 (0.46–1.78)	0.7791	0.73 (0.39–1.38)	0.3436	1.24 (0.55–2.76)	0.6056
	Recessive	187 (0.969)	52 (0.945)	56 (0.933)	0.56 (0.13–2.30)	0.4338	0.45 (0.12–1.65)	0.2433	1.24 (0.26–5.80)	0.7855
*TIMP1 rs4898*	CC	43 (0.223)	19 (0.345)	14 (0.233)	1.00	0.1426	1.00	0.4109	1.00	0.1059
	TC	107 (0.554)	28 (0.509)	28 (0.467)	1.69 (0.85–3.34)		1.24 (0.60–2.59)		1.36 (0.57–3.23)	
	TT	43 (0.223)	8 (0.145)	18 (0.300)	2.38 (0.94–6.01)		0.78 (0.34–1.76)		3.05 (1.04–9.01)	
	Dominant	150 (0.777)	36 (0.655)	46 (0.767)	1.84 (0.96–3.53)	0.0706	1.06 (0.53–2.11)	0.8645	1.73 (0.77–3.92)	0.1839
	Recessive	150 (0.777)	47 (0.855)	42 (0.700)	1.68 (0.74–3.83)	0.1968	1.50 (0.78–2.86)	0.2298	2.52 (0.99–6.39)	0.0452
*TIMP2 rs2277698*	CC	141 (0.731)	39 (0.709)	46 (0.767)	1.00	0.7759	1.00	0.5628	1.00	
	CT	46 (0.238)	15 (0.273)	11 (0.183)	0.85 (0.43–1.68)		1.36 (0.65–2.85)		0.62 (0.26–1.51)	0.3635
	TT	6 (0.031)	1 (0.018)	3 (0.050)	1.66 (0.19–14.20)		0.65 (0.16–2.71)		2.54 (0.25–25.45)	
	Dominant	52 (0.269)	16 (0.291)	14 (0.233)	0.90 (0.46–1.74)	0.7538	1.21 (0.62–2.39)	0.5749	0.74 (0.32–1.71)	0.4825
	Recessive	187 (0.969)	54 (0.982)	57 (0.950)	1.73 (0.20–14.70)	0.5923	0.61 (0.15–2.52)	0.5059	2.84 (0.29–28.17)	0.3401
*TIMP2 rs55743137*	TT	127 (0.658)	32 (0.582)	39 (0.650)	1.00	0.5588	1.00	0.2457	1.00	
	TG	62 (0.321)	22 (0.400)	17 (0.283)	0.71 (0.38–1.32)		1.12 (0.59–2.14)		0.63 (0.29–1.39)	0.2183
	GG	4 (0.021)	1 (0.018)	4 (0.067)	1.01 (0.11–9.33)		0.31 (0.07–1.29)		3.28 (0.35–30.85)	
	Dominant	66 (0.342)	23 (0.418)	21 (0.350)	0.72 (0.39–1.33)	0.3022	0.97 (0.53–1.77)	0.9090	0.75 (0.35–1.59)	0.4524
	Recessive	189 (0.979)	54 (0.982)	56 (0.933)	1.14 (0.13–10.44)	0.9046	0.30 (0.07–1.22)	0.1011	3.86 (0.42-35.62)	0.1863

*p* value multivariable logistic regression.

**Table 8:** Analysis of MMP9 (rs17576 and rs17577) and TIMP2 (rs2277698 and rs55743137) haplotype frequencies with the risk of PE, LOPE and EOPE.

	Frequencies				PE vs. Controls		EOPE vs. Controls		LOPE vs. Controls	
Haplotype	Controls	PE	EOPE	LOPE	OR (95% CI)	*p*	OR (95% CI)	*p*	OR (95% CI)	*p*
*MMP9 loci rs17576 and rs17577*										
*AG*	0.652	0.608	0.645	0.575	0.827 (0.59–1.159)	0.270	0.968 (0.621–1.508)	0.885	0.719 (0.473–1.092)	0.121
*GG*	0.204	0.213	0.190	0.233	1.052 (0.704–1.57)	0.804	0.916 (0.536–1.567)	0.751	1.182 (0.724–1.93)	0.501
*GA*	0.139	0.165	0.154	0.175	1.216 (0.774–1.911)	0.393	1.123 (0.621–2.03)	0.698	1.304 (0.751–2.264)	0.334
*TIMP2 loci rs2277698 and rs55743137*										
*CT*	0.803	0.786	0.781	0.791	0.905 (0.605–1.355)	0.629	0.878 (0.523–1.473)	0.623	0.931 (0.561–1.546)	0.784
*TG*	0.134	0.147	0.154	0.141	1.114 (0.698–1.777)	0.649	1.174 (0.648–2.126)	0.595	1.06 (0.587–1.913)	0.846
*CG*	0.046	0.065	0.063	0.066	1.426 (0.704–2.888)	0.321	1.389 (0.564–3.417)	0.472	1.46 (0.618–3.448)	0.385

Haplotypes below frequency <0.01 are ignored, Pearson’s *p*.

## Data Availability

Not applicable.
